# Peripheral Immune Composition in Alzheimer's Disease: Insights from Multi‐Ancestry RNA‐Sequencing and Cell Deconvolution

**DOI:** 10.1002/alz70856_107423

**Published:** 2026-01-10

**Authors:** Yousef Mustafa, Makaela Mews, Tianjie Gu, Lissette Gomez, Larry D Adams, Takiyah D. Starks, Mario Cornejo‐Olivas, Maryenela Z. Illanes‐Manrique, Concepcion Silva‐Vergara, Briseida E. Feliciano‐Astacio, Goldie S Byrd, Margaret Pericak‐Vance, Jonathan L Haines, Anthony J Griswold, William S Bush

**Affiliations:** ^1^ Systems Biology & Bioinformatics, Department of Nutrition, School of Medicine, Case Western Reserve University, Cleveland, OH, USA; ^2^ John P. Hussman Institute for Human Genomics, University of Miami Miller School of Medicine, Miami, FL, USA; ^3^ University of Miami, Miami, FL, USA; ^4^ Maya Angelou Center for Health Equity, Wake Forest University School of Medicine, Winston‐Salem, NC, USA; ^5^ Neurogenetics Working Group, Universidad Cientifica del Sur, Lima, Peru; ^6^ Universidad Peruana Cayetano Heredia / Center for Global Health, Lima, Peru; ^7^ Neurogenetics Research Center, Instituto Nacional de Ciencias Neurológicas, Lima, Peru; ^8^ Institute of Research, Education and Services in Addiction (IRESA), Universidad Central del Caribe, Bayamon, PR, USA; ^9^ Puerto Rico Alzheimer's disease and Related Disorders Initiative, Caguas, Puerto Rico; ^10^ Wake Forest University School of Medicine, Winston‐Salem, NC, USA; ^11^ Dr. John T. Macdonald Foundation Department of Human Genetics, University of Miami Miller School of Medicine, Miami, FL, USA; ^12^ Cleveland Institute for Computational Biology, Department of Population and Quantitative Health Sciences, Case Western Reserve University, Cleveland, OH, USA; ^13^ Department of Population and Quantitative Health Sciences, Institute for Computational Biology, Case Western Reserve University, Cleveland, OH, USA; ^14^ Department of Population and Quantitative Health Sciences, School of Medicine, Case Western Reserve University, Cleveland, OH, USA

## Abstract

**Background:**

Alzheimer's disease (AD) is marked by neuroinflammation and immune system changes, yet the role of peripheral immune cells remains unclear. Immune function varies by ancestry, influencing disease progression and treatment responses. This study applies high‐resolution immune cell deconvolution of whole blood RNA‐seq data from a multi‐ancestry cohort to characterize AD‐associated immune alterations and ancestry‐specific differences.

**Methods:**

Whole blood RNA‐seq data were generated from non‐Hispanic White (NHW), African‐American (AA), and Hispanic participants, balanced between AD cases and controls. Immune cell proportions were estimated using CIBERSORTx Hi‐Res. The *propeller* R package tested for immune cell differences between AD cases and controls using a main‐effects model adjusted for age, sex, and ancestry. An interaction model assessed ancestry‐specific immune alterations.

**Results:**

The main‐effects model revealed peripheral immune dysregulation in AD, characterized by increased pro‐inflammatory cells and weakened adaptive immunity. AD cases had elevated classical monocytes, cytotoxic monocytes, activated mast cells, M0 macrophages, and neutrophils, along with reductions in non‐classical monocytes, naïve B cells, memory CD8 T cells, resting mast cells, and plasma cells.

The interaction model revealed ancestry‐specific immune alterations. NHW AD cases showed reduced naïve B cells and helper CD4 T cells, with increased cytotoxic monocytes, indicating a pro‐inflammatory shift. Hispanic AD cases exhibited the strongest inflammatory response, with heightened classical monocytes and naïve CD4 T cells but reduced memory CD8 T cells and regulatory CD4 T cells, suggesting excessive innate immune activation and impaired regulation. AA AD cases displayed significant depletion of naïve B cells and an increase in M0 macrophages, indicating adaptive immune deficiencies. Classical monocyte increases were most pronounced in Hispanic AD cases, while regulatory CD4 T cell reductions were more severe in this group than in NHW or AA cases.

**Conclusion:**

This study reveals ancestry‐specific immune dysregulation in AD, emphasizing the role of monocytes and mast cells in disease pathology. The identification of HRH4‐expressing mast cells suggests potential novel inflammatory pathways in AD. These findings highlight varying immune system responses to AD, which may point to different underlying disease processes.

